# miR-30-5p Regulates Muscle Differentiation and Alternative Splicing of Muscle-Related Genes by Targeting MBNL

**DOI:** 10.3390/ijms17020182

**Published:** 2016-01-29

**Authors:** Bo-Wen Zhang, Han-Fang Cai, Xue-Feng Wei, Jia-Jie Sun, Xian-Yong Lan, Chu-Zhao Lei, Feng-Peng Lin, Xing-Lei Qi, Martin Plath, Hong Chen

**Affiliations:** 1 Shaanxi Key Laboratory of Agricultural Molecular Biology, College of Animal Science and Technology, Northwest A&F University, Yangling 712100, Shaanxi, China; zhangbowen361@163.com (B.-W.Z.); caihanfang.cool@163.com (H.-F.C.); weixuefeng.happy@163.com (X.-F.W.); jiajieking@126.com (J.-J.S.); lan342@126.com (X.-Y.L.); leichuzhao1118@126.com (C.-Z.L.); mplath-zoology@gmx.de (M.P.); 2 Department of Animal Husbandry, Bureau of Biyang County of Henan province, Biyang 463700, Henan, China; FengPengLin10@163.com (F.-P.L.); Xing-LeiQi@163.com (X.-L.Q.)

**Keywords:** miR-30-5p, muscle differentiation, MBNL, myotonic dystrophy

## Abstract

MicroRNAs (miRNAs), a class of single stranded, small (~22 nucleotides), non-coding RNAs, play an important role in muscle development. We focused on the role of the miR-30-5p family during bovine muscle development from previous high-throughput sequencing results and analyzed their expression profiles. *MHC* and *MyoG* mRNAs expression as well as their proteins were suppressed in differentiated C2C12 cells, suggesting the importance of miR-30-5p in muscle development. MBNL, the candidate target of miR-30-5p, is an alternative splicing regulation factor. MBNL1 and MBNL3 have opposite effects on muscle differentiation. Our results confirmed that miR-30a-5p and miR-30e-5p repress the expression of MBNL1, MBNL2 and MBNL3, whereas miR-30b-5p inhibits MBNL1 and MBNL2 expression. This provides direct evidence that MBNL expression can be flexibly regulated by miR-30-5p. Previous studies showed that MBNL1 promotes exon inclusion of two muscle-related genes (*Trim55* and *INSR*). Through RNA splicing studies, we found that miR-30-5p had an effect on their alternative splicing, which means miR-30-5p via MBNL1 could be integrated into muscle signaling pathways in which *INSR* or *Trim55* are located. In conclusion, miR-30-5p could inhibit muscle cell differentiation and regulate the alternative splicing of *Trim55* and *INSR* by targeting MBNL. These results promote the understanding of the function of miRNAs in muscle development.

## 1. Introduction

The molecular mechanisms underlying the development of skeletal muscle tissue are the prime focus of research programs aiming at optimizing animal (*i.e*., meat) production. Such agro-economic considerations motivated our present study on the role of microRNAs during muscle differentiation of beef cattle. However, dysfunction of skeletal muscles and especially aberrant muscle differentiation can also result in various diseases, including cancer and diabetes [1,2]. Therefore, identifying as yet unknown factors involved in bovine muscle differentiation not only adds to our general understanding of the mechanisms of mammalian skeletal muscle development and aids breeding programs, but may also have potential applications for research on human diseases.

Skeletal muscle cells (myocytes) of vertebrates are contractile, multinucleated cells that undergo a complex differentiation process during development. The majority of myocytes is derived from the paraxial mesodermal somites: mesodermal progenitor cells proliferate to form myoblasts that undergo a terminal muscle differentiation to form myocytes. The differentiation process starts with the expression of myogenic regulatory factors (MRFs), including myogenic differentiation (MyoD), myogenin (MyoG) and myogenic factor 5 (Myf5), which activate several myocyte enhancer factors [3,4,5]. With the onset of MRF expression, followed by the expression of several muscle-specific genes, cells eventually exit the cell cycle [6,7]. Differentiated myocytes elongate and fuse into multinucleated myotubes, resulting in myofiber formation. Several studies suggest that post-transcriptional regulation also plays an important role in myocyte differentiation. Post-transcriptional regulatory factors identified in previous studies include the K-homology Splicing Regulator Protein (KSRP), CUG-Binding Protein (CUGBP) and Muscleblind-like Protein (MBNL), which are likely involved in the process of muscle differentiation [8,9,10].

Important insights into the process of myogenesis were gained from studies on human diseases like myotonic dystrophy (DM). DM is an autosomal dominant neuromuscular degenerative disease, whose symptoms, among others, include muscle hyper excitability, cardiac conduction defects, skeletal muscle weakening and wasting, and general loss of muscle mass [11]. Two forms of this disease are known: DM1, a trinucleotide repeat disorder, arises when a CTG- repeat in the 3′ untranslated region (3′ UTR) of myotonic dystrophy protein kinase (*DMPK*) gene shows an increased repeat number [12,13]. By contrast, DM2, a tetranucleotide repeat disorder, involves a repeat expansion of a CCTG-repeat in intron 1 of a gene coding for a zinc-finger protein Znf9 [14]. The current model for the occurrence of the DM phenotype is that the (expanded) mutant transcripts accumulate in the nucleus and sequester RNA-binding proteins that are essential for muscle formation and maintenance.

Several proteins binding to, or co-localizing with, the mutant RNAs in DM cells have been identified. In particular, members of muscleblind-like (MBNL) family, including MBNL1, MBNL2, and MBNL3, seem to be involved [15,16,17]. The muscleblind gene was originally described in Drosophila melanogaster, which possesses a single gene [18]. Subsequently, three homologs—muscleblind-like genes *MBNL1*, *MBNL2*, and *MBNL3*—were identified in humans and mice, in which they express mainly in skeletal muscle and the nervous system [15,17,19]. *MBNL* genes code for conserved RNA-binding proteins, which function as alternative splicing factors [16].

MBNL1 was shown to affect the translational inclusion of exon9 of the *Trim55* gene in heart tissue [20], which encodes for a muscle-specific really interesting new gene (*RING*) finger protein. It also contributes to the inclusion of exon11 of the insulin receptor (*INSR*) [21,22]. Importantly, aberrant splicing of *INSR* mRNA determines the fiber type in DM1 and DM2 muscle tissues [23]. MBNL1 and MBNL2 are very similar proteins in the MBNL family and bind with the same consensus sequence. Still, they have specific effects during alternative splicing, and both are required to facilitate some essential splicing events [24]. MBNL1 and MBNL3 have opposing effects on muscle differentiation by regulating the splicing of *Mef2D* beta-exon during terminal muscle differentiation, with MBNL1 as a pro-myogenic factor and MBNL3 as a muscle differentiation inhibitor [9,25,26].

Considerable research has been conducted in an attempt to identify the molecular mechanisms linking the accumulation of mutant RNAs (with extended repeat motifs, see above) to the disease phenotype of DM, and especially the roles of MBNL proteins during pathogenesis have been well explored [15,16,17]. By contrast, studies on the (additional) functions of these three proteins during the process of normal myogenesis are as yet scarce. MBNLs play important roles for muscle differentiation in these cells as well [25], and our present study, focusing on the role of a family of microRNAs (miR-30-5p) on alternative splicing by MBML proteins in cattle, provides novel insights into the fine-tuning of MBML activity during normal muscle tissue development (*i.e*., in the absence of DM).

MicroRNAs (miRNAs) are a class of single stranded endogenous, non-coding, small RNAs of approximately 22 nucleotides’ length. They usually target 3′ UTRs of mRNAs, e.g., for cleaving or translation repression [27]. It has been estimated that the translation of up to 30% of human genes could be potentially regulated by miRNAs [28]. Several studies reported an involvement of miRNAs in myogenic differentiation, especially microRNAs that are specific to striated muscle tissues (myomiR [29]). Muscle-specific miRNAs, such as miR-1, miR-206 and miR-133a, contribute to myoblast differentiation [30,31,32,33]. Through TGF-β signaling, miR-26a and miR-29 could regulate myogenic differentiation [34,35]. By contrast, miR-23a negatively affects muscle differentiation by directly regulating the expression of myosin heavy chain (*MHC*) genes [36]. In addition to these muscle-specific miRNAs, several other miRNAs that non-specifically express in muscle tissues are also involved to myogenesis. miR-181, for example, promotes myoblast differentiation by down-regulating the translation of homeobox protein Hox-A11, which inhibits MyoD expression [37]. miR-24, which has been reported to induce cardiomyocyte hypertrophy, appears to promote skeletal muscle cell differentiation during early stages [38]. Inhibition of miR-27b, targeting the 3′ UTR of *Pax3*, increases satellite cells’ proliferation and delays myogenic differentiation [39]. Even though many miRNAs participating in muscle development have been identified, their number is negligible compared to that of miRNA in miRBase containing 30,424 mature miRNAs [40], and are likely lead to the verification of the involvement of additional miRNAs during myogenesis.

During the previous research about high-throughput sequencing on miRNA in skeletal muscle of Chinese cattle, the miRNA family miR-30-5p (miR-30a-5p, miR-30b-5p and miR-30e-5p) was detected and was predicted to target MBNL mRNA and, thus, to play a role in myogenesis [41]. These miRNAs play an important role in the development of cardiomyocytes and vascular smooth muscle cell, and are involved in cell myocardial hypertrophy [42,43,44], and, thus, could also be involved in the development of skeletal muscle tissue. The aim of our present study was to investigate the function of miR-30-5p in the regulation of myogenesis. We tested the hypothesis that members of the miR-30-5p family regulate myogenesis by targeting members of the MBNL family and, thus, facilitate alternative splicing of muscle-related genes.

## 2. Results

### 2.1. The Expression Profile of miR-30-5p in Different Tissues

**Figure 1:**
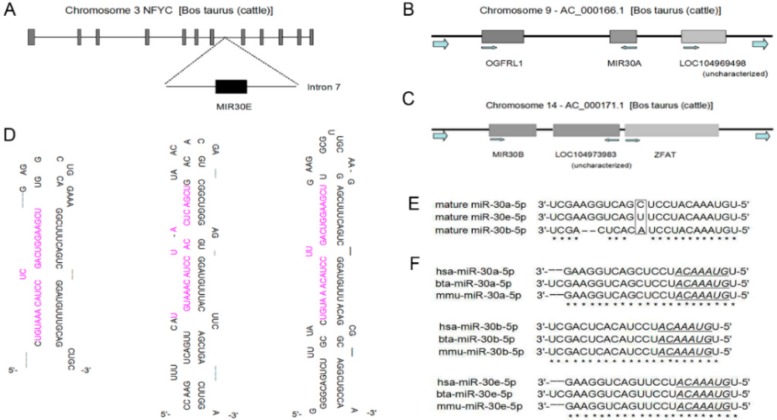
The analysis of miR-30-5p. (**A**) Genomic structure of NFYC gene and the primary transcription unit (MIR30E) for bta-miR-30e-5p in the intron7; (**B**) Genomic localization of MIR30A that processes bta-miR-30a-5p; (**C**) Genomic localization of MIR30B that processes bta-miR-30b-5p; (**D**) The stem-loop structures of bovine pre-miR-30 (pre-miR-30a, pre-miR-30b and pre-miR-30e). The mature cattle miR-30-5p (miR-30a-5p, miR-30b-5p and miR-30e-5p) from 5′ arm of of the hairpin precursors are labeled in pink; (**E**) Alignment of mature miR-30-5p, it showed the conserved match to the mature sequences. The bases in box is different among the three miRNA sequences. The stars stand for conservatism among different sequences; (**F**) The similarity analysis of miR-30-5p in human, cattle and mouse. The sequences underlined represent the seed sequence of miR-30-5p. The stars stand for conservatism among different sequences.

**Figure 2:**
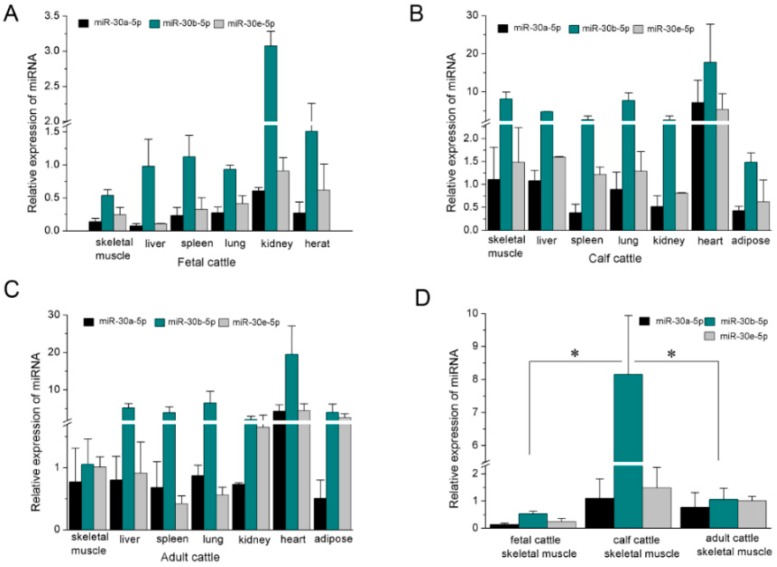
Expression profile of miR-30-5p in tissues of different stage. (**A**-**C**) RT-qPCR detection of mature miR-30-5p in fetal (**A**), calf (**B**) and adult (**C**) cattle tissues. The expression level was normalized to U6 and relative to miR-30e-5p expression in muscle of adult cattle; (**D**) The differences of miR-30-5p expression in skeletal muscle among fetal, calf and adult cattle. * *p* < 0.05; Error bars indicate SD (Standard Deviation) (*n* = 3).

Although bovine miR-30a-5p, miR-30b-5p and miR-30e-5p are located on the different chromosomes (Figure 1A–C), the mature miRNAs from the 5′ arm of the hairpin precursors are conserved from mouse to human species (Figure 1D–F). To address the function of bovine miR-30-5p, we first detected the expression patterns in different tissues of fetal, calf, and adult Qinchuan cattle by RT-qPCR analysis. High expression of mature miR-30-5p was found in heart and lung tissue (Figure 2A–C). Heart muscle is one of three types of muscle tissues and the lung is rich in smooth muscle fibers. Skeletal and heart muscle comprise striated muscle cells that originate from the mesodermal embryonic layer. Although the expression of miR-30a-5p and miR-30e-5p in skeletal muscle tissue was not significantly different to that in most other tissues, both miRNAs were among the top 10 most frequently detected known miRNAs in bovine skeletal muscles [45], implying their important roles in muscle tissue development. Additionally, we found that the expression of mature miR-30b-5p demonstrated a great difference in skeletal muscle during different stages of Qinchuan cattle development (Figure 2D), suggesting that miR-30b-5p might be developmentally expressed in muscle cells. Thus, because the expression profile suggested that miR-30-5p might have an important role in muscle development, it was selected as the candidate miRNA for further research about muscle differentiation.

### 2.2. miR-30-5p Inhibits Myogenic Differentiation

**Figure 3:**
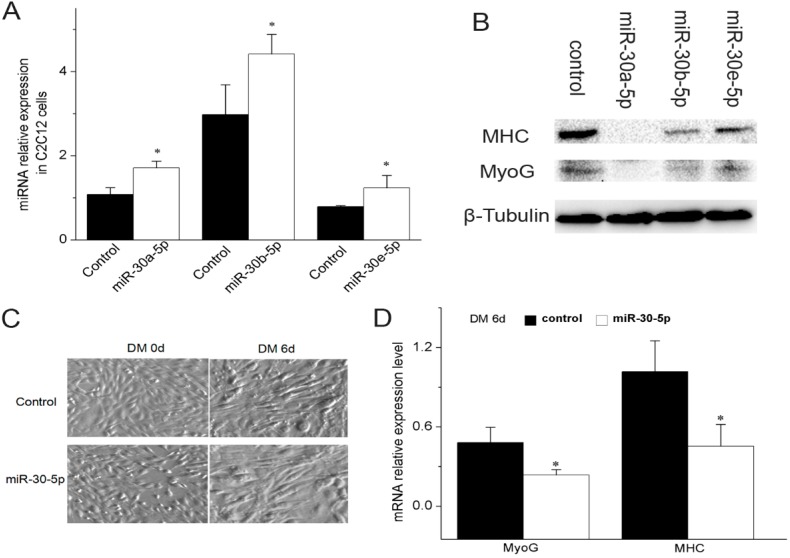
Effect of miR-30-5p on differentiation of C2C12 myoblasts. (**A**) Constructs and expression of miR-30-5p. RT-qPCR detection of mature miR-30-5p, using RNA prepared from C2C12 cells transfected with the expression constructs of miR-30-5p during the differentiation, confirming proper processing of miR-30-5p. The cells transfected with pcDNA3.1(+) as the control; (**B**) Western blot detection for MHC and MyoG proteins in the differentiated C2C12 cells respectively transfected miR-30a-5p, miR-30b-5p and miR-30e-5p constructs for six days in differentiation medium (DM). β-Tubulin was used as the loading control; (**C**) The C2C12 cells cultivated in DM (magnification 10×); (**D**) The expression levels of MHC and MyoG in C2C12 cells co-transfected with equivalent amount of miR-30a-5p, miR-30b-5p and miR-30e-5p were detected by RT-qPCR at 6 days after transfection in DM. control represents the C2C12 cells not transfected by miR-30-5p. Asterisks indicate significant differences. * *p* < 0.05; Error bars indicate SD (*n* = 3). Days (d) indicate the time the cells were in the differentiation medium. The expression level was normalized to GAPDH.

Because mature miR-30-5p sequences between bovine and murine are so highly conserved (Figure 1F), we chose mouse C2C12 cells as the experimental model. To study the possible function of miR-30-5p in myogenesis, we first overexpressed miR-30a-5p, miR-30b-5p and miR-30e-5p in C2C12 cells and detected the mature miR-30-5p expression level by RT-qPCR. Significant difference was observed between C2C12 cells transfected with pcDNA3.1(+) containing pre-miR-30-5p fragments and cells with empty pcDNA3.1(+) (Figure 3A), indicating that the constructs successfully generated the mature miR-30-5p. The advantage of these constructs is the stability of processing mature miR-30-5p. After the transfection of miR-30a-5p, miR-30b-5p and miR-30e-5p individually into the C2C12 cells, the protein expression level of myosin heavy chain (MHC) and MyoG were examined. As shown in Figure 3B, at six days of differentiation, the MHC and MyoG protein expression levels in C2C12 cells transfected with pcDNA3.1(+) harboring pre-miR-30-5p were significantly lower than in the control C2C12 cells. In Figure 3C, we observed a phenotypic change of C2C12 during differentiation. The mRNA expression analysis showed that there was a lower level of MyoG and MHC in the C2C12 co-transfected with miR-30-5p than in the control C2C12 at six days of differentiation (Figure 3D). Thus, the expression data of myogenic markers indicated that miR-30-5p could suppress the differentiation of C2C12 myoblasts.

### 2.3. miR-30-5p Directly Targets MBNL Family

#### 2.3.1. miR-30-5p Directly Targets MBNL1

**Figure 4:**
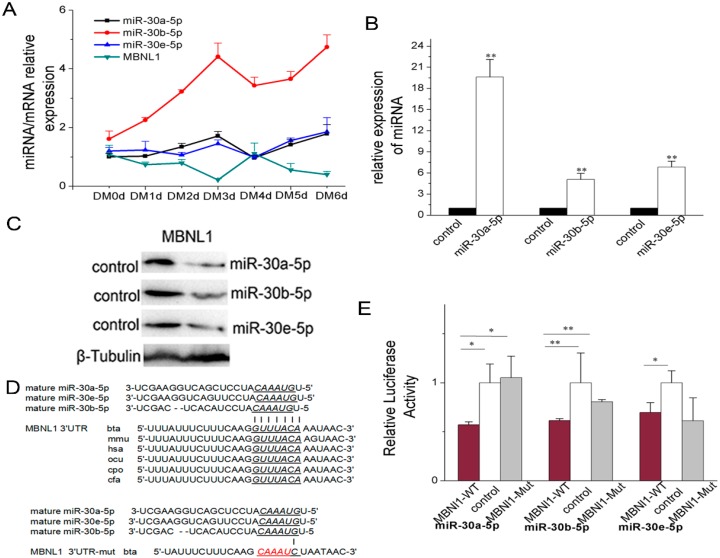

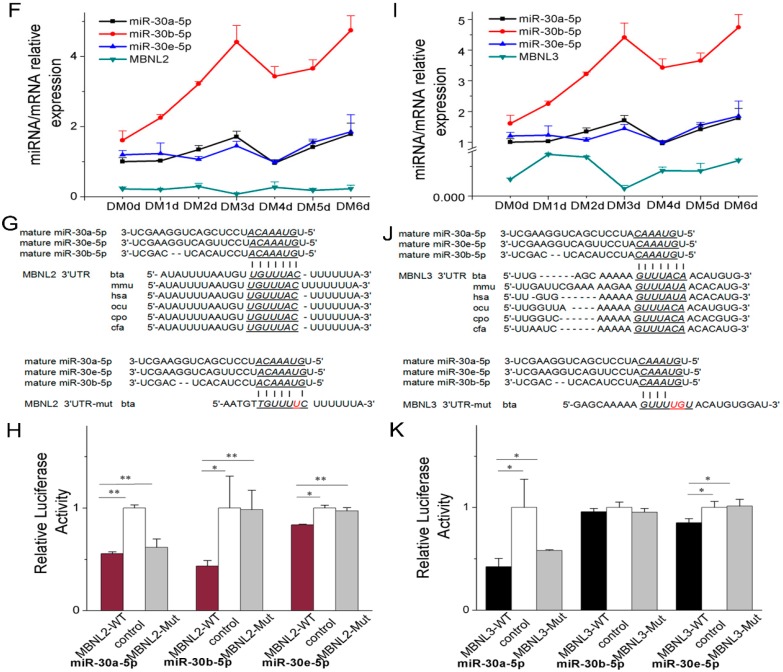
miR-30-5p directly targets MBNL. (**A**) The expression level of MBNL1 and miR-30-5p (miR-30a-5p, miR-30b-5p and miR-30e-5p) in the C2C12 cells co-transfected with miR-30-5p at DM 0, 1, 2, 3, 4, 5 and 6 days; (**B**) Constructs and expression of miR-30-5p. RT-qPCR detection of mature miR-30-5p, using RNA prepared from HEK293T cells transfected with the expression constructs of miR-30-5p, confirming proper processing of miR-30-5p. The cells transfected with pcDNA3.1(+) as the control; (**C**) Western blot analysis of MBNL1 protein levels regulated by miR-30-5p in the HEK293T cells respectively transfected with miR-30a-5p, miR-30b-5p and miR-30e-5p. Control was the cells without any treatment. β-Tubulin was used as the loading control; (**D**) Sequence alignment of potential binding site of miR-30-5p in the 3′ UTR of MBNL1, MBNL2 and MBNL3. Cattle wild type (MBNL1-WT) is upper and mutated type (MBNL2-Mut) is lower. The potential binding sites and the seed sequences of miR-30-5p were showed with potential binding sites underlined. The red font stands for the mutated bases in the potential binding site; (**E**) Luciferase assays for the direct evidence of miR-30-5p targeing MBNL1. miR-30a-5p, miR-30b-5p and miR-30e-5p were respectively transfected into HEK293T cells with the luciferase reporter constructs harbouring potential binding sites of miR-30-5p (MBNL1-WT) or the luciferase reporter constructs harbouring mutant potential binding sites of miR-30-5p (MBNL1-Mut); (**F**–**H**) miR-30-5p directly targets MBNL2; (**I**–**K**) miR-30-5p directly targets MBNL3. As a control, the empty luciferase reporter vector (control) was co-transfected into HEK293T cells with miR-30-5p. Asterisks indicate significant differences. * *p* < 0.05; ** *p* < 0.01. Error bars indicate SD (*n* = 3).

TargetScan6.2 was used to predict the target genes for bovine miR-30-5p. MBNL1 was selected as a candidate owing to its roles in muscle differentiation. The prediction results revealed MBNL1 to have potential sites recognized by 7~8 mer seed sequences of miR-30-5p (miR-30a-5p, miR-30b-5p and miR-30e-5p) (Figure 4D), and that the potential sites were conservative in the 3′ UTRs of mammalian MBNL1 (Figure 4D). The co-transfection experiment of miR-30-5p and the non-transfected experiment showed that there was a negative correlation between the miR-30-5p expression levels and MBNL1 (Figure 4A and Figure S1), suggesting that miR-30-5p could likely regulate MBNL1 expression. Before conducting the following experiments, we had proven the ability of the miR-30-5p constructs to generate mature miRNA in HEK293T cells (Figure 4B). To obtain direct evidence that MBNL1 3′ UTRs are targets of miR-30-5p, we cloned the 3′ UTRs of the *MBNL1* gene, including the miR-30-5p recognition sites, and inserted them downstream of the luciferase gene in the pGL3-control luciferase reporter vector. Mutant constructs were also generated (Figure 4D lower). Compared with HEK293T cells transfected with empty luciferase reporter vector, the luciferase activity in HEK293T cells transfected with the MBNL1 3′ UTR (mixed with miR-30a-5p, miR-30b-5p and miR-30e-5p, respectively) were severely repressed (Figure 4E). Point mutation analyses showed that the miR-30-5p seed sites had no ability to target the mutated target sites of 3′ UTRs, with the exception miR-30e-5p (Figure 4E). Western blot analysis confirmed that the endogenous MBNL1 proteins in HEK293T cells were particularly and significantly attenuated by miR-30-5p (Figure 4C). These results verified that MBNL1 was the target gene regulated by miR-30-5p.

#### 2.3.2. miR-30-5p Directly Targets MBNL2 and MBNL3

*MBNL2* and *MBNL3* were also predicted to be target genes for bovine miR-30-5p, according to TargetScan6.2. The prediction results revealed *MBNL2* and *MBNL3* have potential sites recognized by 7~8 mer seed sequences of miR-30-5p (Figure 4G,J), and that the potential sites were conservative in 3′ UTRs of *MBNL2* in mammal (Figure 4G), but not in *MBNL3* (Figure 4J). The co-transfection experiment of miR-30-5p and the non-transfected experiment showed a negative correlation between the miR-30-5p expression levels and *MBNL2* and *MBNL3* expression (Figure 4F,I, Figures S2 and S3), suggesting that miR-30-5p could be likely to regulate the expression of *MBNL2* and *MBNL3*. In the cells transfected with the luciferase reporter vector containing *MBNL2* 3′ UTR targeting the seed sequences of miR-30a-5p, miR-30b-5p and miR-30e-5p, the luciferase activity was significantly suppressed, which was in contrast with results from the control cells (Figure 4H). Meanwhile, co-transfection of *MBNL3* 3′ UTR with miR-30a-5p and miR-30e-5p resulted in reduced luciferase activity (Figure 4K). Also, point mutation analyses showed that the miR-30-5p seed sites had no capability of targeting the mutated target sites of the 3′ UTRs (Figure 4H,K). Taken together, these data indicated that miR-30a-5p, miR-30b-5p and miR-30e-5p directly target the 3′ UTR of *MBNL2*, whereas *MBNL3* is a direct target gene of miR-30a-5p and miR-30e-5p only.

### 2.4. MBNL1 Promotes Muscle Differentiation

**Figure 5:**
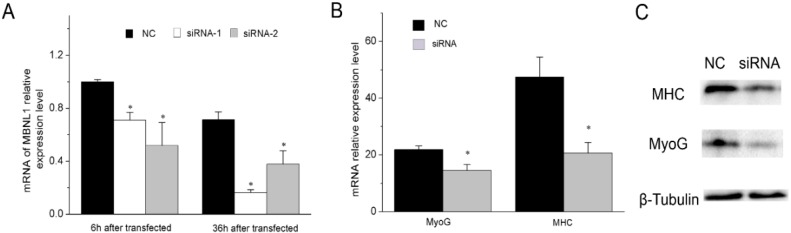
MBNL1 represses muscle differentiation (**A**) The mRNA expression level of MBNL1 in C2C12 cells transfected with siRNA (siRNA-1 and siRNA-2); (**B**) The mRNA expression level of MyoG and MHC in C2C12 cells in differentiation medium for 36 h after transfection; The expression level was normalized to GAPDH; (**C**) Western blot detection for MHC and MyoG proteins after transfection with siRNA-1 into C2C12 cells in differentiation medium for two days. β-Tubulin was used as the loading control. The cells transfected with NC were control. Asterisks indicate significant differences. * *p* < 0.05; Error bars indicate SD (*n* = 3).

Although a previous study has suggested that MBNL and its mammalian homolog are required in muscle cell differentiation [9], the specific function of MBNL1 in the process is still unknown, and requires more experimental investigation. In this paper, we validated the effect of siRNA-mediated MBNL1 knockdown on muscle cell differentiation. siRNA-1 and siRNA-2 against MBNL1 were transfected respectively into C2C12 cells, resulting in a decrease of MBNL1 mRNA expression (Figure 5A). We selected siRNA-1 for transfection into C2C12 cells to study both the mRNA and protein expression levels of MyoG and MHC. The results showed that both the mRNA and protein expression of MyoG and MHC were significantly inhibited (Figure 5B,C), validating the fact that MBNL1 promotes muscle differentiation. Hence, we can conclude that miR-30-5p regulates muscle differentiation through MBNL1 repression.

**Figure 6:**
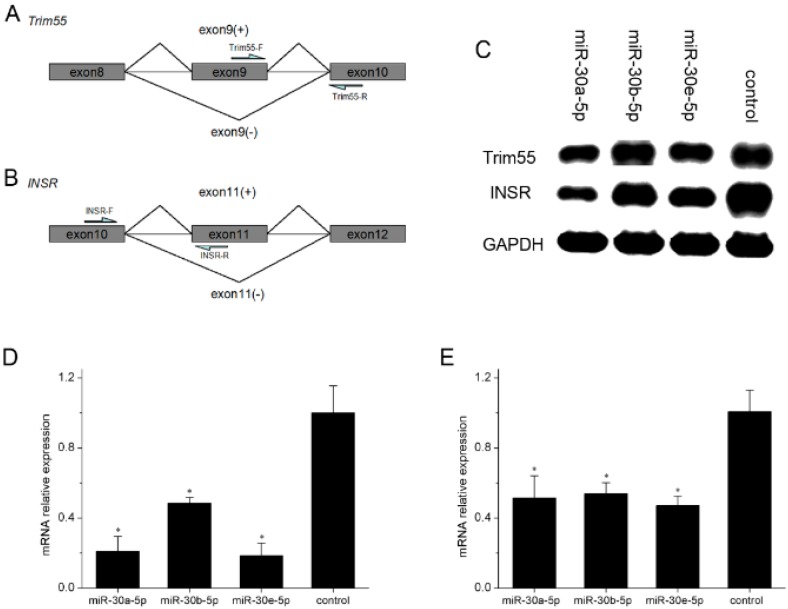
Effect of miR-30-5p on alternative splicing of Trim55 and INSR (**A**,**B**) The primers for the alternative splicing of Trim55 and INSR; (**C**) Total RNA during the six days of C2C12 cells’ differentiation was extracted, and cDNA was amplified. The cDNA was used to produce the Trim55(exon9+) and INSR(exon11+), which were detected by gel electrophoresis; (**D**) Relative expression levels of Trim55(exon9+) at six days in differentiation medium; (**E**) Relative expression levels of and INSR(exon11+) at six days in differentiation medium. DM six day control represents the C2C12 cells not transfected by miR-30-5p. Asterisks indicate significant differences. * *p* < 0.05; the expression level was normalized to GAPDH. Error bars indicate SD (*n* = 3).

### 2.5. miR-30-5p Regulates the Alternative Splicing of Trim55 and INSR by MBNL1

MBNL1 is an alternative splicing regulation factor. To obtain further information on how miR-30-5p downregulates the MBNL1 level, we determined the splice pattern of Trim55 and INSR (Figure 6A,B), which are regulated by MBNL1. We individually transfected constructs expressing miR-30a-5p, miR-30b-5p and miR-30e-5p into C2C12 cells during differentiation. Total RNA was extracted for reverse transcription. The transcripts Trim55(exon9+) and INSR(exon11+) were produced from the cDNA obtained from the total RNA, which were detected by gel electrophoresis (Figure 6C). qPCRs were conducted for Trim55(exon9+) and INSR(exon11+). As shown in Figure 6D,E, the expression levels of Trim55(exon9+) and INSR(exon11+) in C2C12 cells transfected with miR-30-5p were obviously lower than that in control C2C12 cells, indicating that miR-30-5p could regulate the alternative splicing of the target genes of MBNL1, providing more evidence that miR-30-5p could repress MBNL1 protein expression.

## 3. Discussion

According to our previous research on its expression in muscle tissue, miR-30-5p (miR-30a-5p, miR-30b-5p and miR-30e-5p) is a muscle-related miRNA [41]. Several studies on miR-30 have been reported recently. Under Angiotensin II induction, miR-30a in cardiomyocytes facilitates myocardial hypertrophy through excessive autophagy by targeting beclin-1 (an autophagy-related gene) [43]. NF-κB-mediated miR-30b targets Bcl-2 to control cardiomyocytes cell death. Vascular calcification is currently considered to be the process that recapitulates skeletal bone formation, and miR-30b and miR-30c can promote vascular smooth muscle cell calcification [42,44]. Aside from these findings, research about the function of miR-30 in skeletal muscles has rarely been carried out. In this study, we first confirmed the effect of miR-30-5p on skeletal muscle differentiation. The expression profiling analysis showed evidence that miR-30-5p might play a crucial role in muscle development. Skeletal muscle functions to control movements and support respiration [1]. The process of myofibers formation from muscle progenitor cells involves many transcription factors, such as MyoG and MHC [46,47,48]. miR-30-5p was confirmed to repress both the mRNA and protein expression of MHC and MyoG, which provided evidence that miR-30-5p repressed muscle differentiation. However, the mechanism of how miR-30-5p regulates myogenic process is not clear.

Ever since the MBNL gene was initially reported in Drosophila melanogaster [18], much attention has been focused on elucidating the impact of the MBNL proteins in Myotonic Dystrophy. Some recent studies have confirmed that the MBNL proteins also function in normal cells and they have an effect on muscle differentiation [25]. The prediction of TargetScan6.2 showed that the MBNL family of genes are the potential direct targets of miR-30-5p, and our data verified the prediction. Based on our RNAi experiments and previous study on the roles of MBNL1 and MBNL3 in muscle differentiation [25,49], we concleded that miR-30-5p regulates muscle differentiation through directly targeting the MBNL genes.

All miR-30a-5p, miR-30b-5p and miR-30e-5p could regulate MBNL1 and MBNL2, which are highly similar proteins in the MBNL family. As alternative splicing regulatory factors, they not only have unique contributions to alternative splicing, but are also dually-required in some splicing events [24]. Through the myocyte enhancer factors (MEFs), MBNL1 and MBNL2 promote MBNL2 exon5 exclusion [50,51], and MBNL1 also promotes MBNL2 exon7 inclusion that determines MBNL1 dimerization [52]. Whereas MBNL2 exon5 exclusion is able to compensate for the lack of MBNL1, MBNL2, exon5 inclusion is not [24]. These previous studies strongly suggested that all three members of miR-30-5p jointly participate in the auto-regulation and cross-regulation of MBNL1 and MBNL2, which means that miR-30-5p may serve an important role in the accurate modulation of MBNL1 and MBNL2 function. Additionally, exon5 and exon7 of MBNL1 are mainly distributed in early differentiation stages [53,54], which suggests that miR-30-5p regulates muscle differentiation possibly by regulating alternative splicing transcripts of MBNL1 and MBNL2. Besides this, we also found that the members of miR-30-5p showed differences in regulating the MBNL members. miR-30b-5p could repress MBNL1 and MBNL2 expression, but had no effect on MBNL3 expression, which may be important to the MBNL1/MBNL3 pair that controls muscle differentiation [26]. When the cells did not differentiate, MBNL3 was highly expressed, inhibiting Mef2D β-exon inclusion. Mef2D that includes β-exon is able to activate MBNL1, whereas Mef2D without β-exon is inactive. During differentiation, MBNL3 reduces concomitantly with an increase in Mef2D including β-exon. We therefore concluded that miR-30b-5p and MBNL3 protein may have similar functions on MBNL1, suggesting that miR-30b-5p is integrated into the MBNL1/MBNL3 pair model to compensate for MBNL3. However, the reason why miR-30b-5p has a different function on MBNL3 compared with miR-30a-5p and miR-30e-5p is not known, We surmise that it might be as a result of the different bases among these mature miRNAs (Figure 1E), which means that more work should be done to further clarify the details.

The PI3K/AKT (protein kinase B) pathway and ERK pathway are two muscle signaling pathways [21,55]. In the PI3K/AKT pathway, activated AKT promotes muscle growth. In the ERK pathway, ERK, a kinase of the MAPK family, phosphorylates a set of substrates, (among them the transcription factor MEF2) to regulate muscle development. Above all, the two pathways are initiated by the binding of insulin to INSR, which is mainly expressed in liver tissue, adipose tissue, and skeletal muscle. INSR has two isoforms: IR-A (lacking exon11) and IR-B (including exon11). IR-B is reported to mediate cell differentiation, and IR-A is often detected in cancer cells. Moreover, the abnormally high IR-A/IR-B ratio in muscle cells may greatly contribute to the development of myotonic dystrophy disease [21]. We observed that the C2C12 cells transfected with miR-30-5p had a reduced mRNA expression of IR-B, confirming that miR-30-5p regulates the alternative splicing of INSR, which suggests that miR-30-5p may control muscle differentiation through the PI3K/AKT and ERK pathway. In addition, the Trim55 gene encodes a muscle-specific protein involved in sarcomere assembly [56]. As expected, the mRNA expression of Trim55 including exon9 decreased in this study, suggesting that miR-30-5p is likely to regulate more muscle-related genes like Trim55 to act on muscle differentiation. Meanwhile, both MBNL1 and MBNL2 are RNA binding proteins, which are alternative splicing factors [16]. Decreasing MBNL1 levels promoted INSR exon11 exclusion [22], and the loss of MBNL1 resulted in Trim55 exon9 exclusion [20]. Therefore, we reasonably concluded that miR-30-5p directly targets MBNL for regulating muscle differentiation through muscle signaling pathways.

## 4. Materials and Methods

### 4.1. Animals and Expression Profile Analysis

Use of animals and the procedures performed in this study were approved by Northwest A&F University Institutional Animal Care and Use Committee.

Samples of different tissues (heart, liver, spleen, lung, kidney and skeletal muscle) were collected from fetal (gestational age at 24 months), calf (new born) and adult Chinese Qinchuan cattle (24 months) (*n* = 3, respectively). Total RNA was extracted and reverse transcript were performed. Sufficient cDNA was prepared to perform the qPCR for all the tissues from different stage. Primers listed in the Table S1 for miR-30-5p and reference gene *U6* were designed based on Bos taurus sequences using Beacon Designer 7.9.

### 4.2. Constructs’ Generation

miRNA constructs that express miR-30-5p including miR-30a-5p, miR-30b-5p and miR-30e-5p were conducted, using the overexpression vector pcDNA3.1(+). The pre-miR-30-5p were amplified from the genome of Chinese Qinchuan cattle using the primers in Table S2. The fragments containing the pre-miR-30-5p were then digested by *Hin*dIII and *Kpn*I restriction enzyme (TakaRa; Tokyo, Japan) and inserted into the pcDNA3.1(+) vector with T4 DNA ligase (TakaRa; Japan), and confirmed by sequencing.

Luciferase reporter vectors were constructed, with the 3′ UTRs of *MBNL* (*MBNL1, MBNL2* and *MBNL3*) cloned into the *Xba*I site in luciferase reporter vector pGL3-control (primers listed in Table S2). Moreover, overlap PCR was carried out to generate the mutations in target sites of *MBNL* 3′ UTRs recognized by miR-30-5p, using two pairs of primers, two of them harboring the mutations (Primers listed in Table S3). Then, the mutated 3′ UTRs were digested and subcloned into the XbaI site of pGL3-control vector.

### 4.3. Cell Culture

The HEK293T cells (ATCC; Manassas, VA, USA) were maintained in a 6 cm culture dish containing cell culture medium, high-glucose DMEM (Hyclone; Beijing, China) supplemented with 10% fetal bovine serum (FBS) (Hyclone; Beijing, China) and antibiotic (100 U/mL penicillin and 0.1 mg/L streptomycin) at 37 °C with 5% CO_2_. The time interval was one day for cell culture.

Mouse C2C12 myoblasts (ATCC; USA) were maintained in high-glucose DMEM supplemented with 10% fetal bovine serum (GM) and cultured at 37 °C with 5% CO_2_. Myogenic differentiation was induced by DMEM with 2% horse serum (DM) (Hyclone; Beijing, China) after cells reached a confluence of 70%–80%. The time interval was one day for replacing culture medium before the end of the checkpoint.

### 4.4. Transfection

To test whether the miR-30-5p constructs could generate mature miRNA, we transfected the recombinational vectors pcDNA3.1(+) containing pre-miR-30-5p in HEK293T cells. When 90% of the area of the bottom of the 6 cm culture dish was covered, the cells were seeded in 6-well plates and grown for 24 h. Then, HEK293T cells were transfected with 2.0 μg of pcDNA3.1(+) containing pre-miR-30-5p constructs at a confluence of 90% using Lipofectamine 2000 (Invitrogen; Grand Island, NY, USA) according to the manufacturer’s protocol. The cells were then cultured in the serum-free and antibiotic-free medium. After 6 h, the cells were cultured in the medium (DMEM with 10% FBS) for an additional 24–48 h before assay.

To confirm the effect of miR-30-5p on muscle differentiation, the mixture with an equivalent amount of constructs expressing miR-30a-5p, miR-30b-5p and miR-30e-5p would be transfected into the C2C12 cells cultured in the 6-well plates. When the confluence reached 80%, the mixed constructs were transfected into the C2C12 cells cultured in the DM medium for 7 days. The cells were collected at one day intervals. A group of C2C12 cells without any treatment was used as a control.

### 4.5. Real-Time Quantitative PCR (RT-qPCR)

Total RNA, including miRNA, was extracted from cells using Trizol reagent from TakaRa. For qPCR analysis, 500 ng total RNA was reverse-transcribed with PrimeScript RT reagent Kit (TakaRa; Japan) with or without specific inverse transcription stem-loop primers designed by us according to the manufacturer’s protocol, and then SYBR Green PCR Master Mix Reagent Kit (TakaRa; Japan) was used for qPCR. *U6* and *GAPDH* were used as internal control. The qPCR parameters were as follows: cycle 1, 95 °C for 3 min; cycle 2, 95 °C for 5 s, 54 °C for 30 s for 40 cycles. The 2^ΔΔ*C*t^ method was used for calculating the fold change of expression of the transcript/miRNA. The gene-specific primers listed in Table S4 for *MBNL1*, *MBNL2*, *MBNL3*, *Trim55*, *INSR*, *MHC*, *MyoG* and *MyoD* were designed by Beacon Designer 7.9.

### 4.6. miRNA Target Prediction

Mature sequences of bta-miR-30a-5p (Accession, MIMAT0003841), bta-miR-30b-5p (MIMAT0003547) and bta-miR-30e-5p (MIMAT0003799) were downloaded from miRBase and the 3′ UTR sequence of bovine MBNL1 (Accession，XM_005201835), MBNL2 (NM_001099710) and MBNL3 (XM_005227518) were downloaded from NCBI. The bta-miR-30a-5p, bta-miR-30b-5p and bta-miR-30e-5p targets predicted by the Targetscan6.2 algorithms [57].

### 4.7. Luciferase Activity Assay

Luciferase activity assays were performed using the Dual-Luciferase reporter assay System (Promega; Madison, AL, USA). The HEK293T cells were seeded into 12-well plates and were cultured in DMEM supplemented with 10% FBS. The cultures were changed for serum-free medium prior to transfection with mixed vectors composed of 200 ng Firefly Luciferase reporter combinational constructs, 50 ng Ranilla Luciferase reporter vectors and 2.5 μg pcDNA3.1(+) containing pre-miR-30-5p constructs, using Lipofectamine 2000 (Invitrogen; Grand Island, NY, USA) according to manufacturer’s protocol. After 6 h, the transfection reagent was replaced with culture medium (DMEM with 10% FBS), which were maintained at 37 °C for 24–48 h. Cells were then harvested using 250 µL Passive Lysis Buffer (PLB) and the culture plates was rocked at room temperature for 15 min. The lysate was transferred to a tube. Up to 20 µL of cell lysate was transferred into 96-well plates in triplicate containing 100 µL/well Luciferase Assay Reagent II (LARII) for luciferase activity and was mixed by pipetting 2 or 3 times. Then, the 96-well plates were put in MPPC luminescence analyzer (HAMAMATSU; Beijing, China). For the Renilla luciferase activity assay, 100 µL Stop & Glo^®^ Reagent was added to the 96-well plates with mixed LAR II and cell lysate. Luciferase activities were normalized against Renilla Luciferase activity.

### 4.8. Western Blot

Proteins were extracted from the HEK293T transfected with the mixture of equivalent amount of miR-30-5p constructs, using RIPA buffer (Solarbio; Beijing, China) containing 1 mM PMSF (Solarbio; Beijing, China). Then, SDS-polyacrylamide gel electrophoresis (SDS-PAGE) was performed in the 8% or 12% polyacrylamide gels with 20 μg proteins per lane. After this, the proteins in the gels were transferred onto the PVDF membrane that then were blocked with 5% skim milk in Tris Buffered Saline with Tween (TBST) buffer (0.15 M NaCl, 1 M Tris–HCl pH 8.0, 0.05% Tween-20) for 2 h at room temperature. After incubation with primary antibody against MBNL1 (dilution 1:1000; ab108519; Abcam, Shanghai, China), MyoG (dilution 1:1000; ab124800; Abcam, Shanghai, China), MHC (dilution 1:1000; ab24648; Abcam, Shanghai, China) at 4 °C overnight, the PVDF membrane were washed in TBST buffer and incubated with secondary antibody for the anti-immune rabbit IgG (dilution 1:4000; LK2001; Sungene Biotech, Tianjin, China) conjugated with HRP. Antibody reacting bands were detected by means of ECL detection reagents. The β-tubulin was used as internal control, whose primary antibody (dilution 1:1000; KDM9003; Sungene Biotech, Tianjin, China) and secondary antibody for anti-immune mouse IgG-HRP (dilution 1:4000; LK2003; Sungene Biotech, Tianjin, China) were purchased from the Tianjin Sungene Biotech.

### 4.9. Transfection of siRNA

To study the role of MBNL1 in muscle differentiation, two custom siRNA duplexes were designed and synthesized by GenePharma Company for RNAi against mouse MBNL1. Those two sequences were as follows: siRNA-1: CAUAAUAUCUGCCGAACAUTT; siRNA-2: ACAAGUAUGUUACCCAGAUTT). For siRNA transfection, C2C12 cells were cultured in 24-well plates and incubated at 37 °C with 5% CO_2_ overnight. Then, 100 nM siRNA was transfected using Lipofectamine 2000 (Invitrogen; Grand Island, NY, USA). Transfected cells were incubated at 37 °C in 5% CO_2_, and respectively harvested after 6 and 36 h for extracting RNA and Western blot. For controls, we transfected Negative control (NC) siRNA into C2C12 cells.

### 4.10. Statistical Analysis

The data in this study were obtained from at least three independent experiments. Quantitative data are expressed as the means ± SD. Statistics are calculated with SPSS statistics v17.0 software. Student’s *t*-test was used to determine significant differences among means.

## 5. Conclusions

In summary, we observed that miR-30-5p affects muscle differentiation and that MBNL1 promotes muscle differentiation, which demonstrated that the *MBNL* family members are the direct target genes of miR-30-5p, and confirmed that miR-30-5p could regulate alternative splicing of the *INSR* and *Trim55* genes. We concluded that miR-30-5p directly targets MBNLs, through muscle signaling pathways, to regulate muscle differentiation. Above all, our findings provide better knowledge about the flexibility in regulating the biological pathway of muscle development and differentiation, and may even contribute to the prevention of the pathogenesis of myotonic dystrophy.

## Supplementary Materials

Supplementary materials can be found at http://www.mdpi.com/1422-0067/17/2/182/s1.
