# Supplementary material for: miR-30-5p Regulates Muscle Differentiation and Alternative Splicing of Muscle-Related Genes by Targeting MBNL
Source: Int J Mol Sci. 2016 Jan 29;17(2):182. doi: 10.3390/ijms17020182 (PMC4783916; doi:10.3390/ijms17020182)
Supplement: Supplementary file 1 [file ijms-17-00182-s001.pdf]

# Supplementary Materials: miR-30-5p Regulates Muscle Differentiation and Alternative Splicing of Muscle-Related Genes by Targeting MBNL

**Bo-Wen Zhang, Han-Fang Cai, Xue-Feng Wei, Jia-Jie Sun, Xian-Yong Lan, Chu-Zhao Lei, Feng-Peng Lin, Xing-Lei Qi, Martin Plath and Hong Chen**

**Table S1.** Primers for the real-time PCR for miR-30-5p.

| Name of Primers | Sequence of Primers (5′-3′) | Amplicon Size |
|---|---|---|
| Stem-loop RT-miR-30a-5p | gtcgtatccagtgcagggtccgaggtattcgcactggatacgacagcttc | |
| miR-30a-5p-F | GGCGTGTAAACATCCTCGACTG | |
| miR-30a-5p-R | GTGCAGGGTCCGAGGT | 62 bp |
| Stem-loop RT-miR-30b-5p | gtcgtatccagtgcagggtccgaggtattcgcactggatacgacagctga | |
| miR-30a-5p-F | GGCGTGTAAACATCCTACACTC | |
| miR-30a-5p-R | GTGCAGGGTCCGAGGT | 60 bp |
| Stem-loop RT-miR-30e-5p | gtcgtatccagtgcagggtccgaggtattcgcactggatacgacagcttc | |
| miR-30e-5p-F | GGCGTGTAAACATCCTTGACTG | |
| miR-30e-5p-R | GTGCAGGGTCCGAGGT | 62 bp |
| U6-F | GCTTCGGCAGCACATATACTAAAAT | |
| U6-R | CGCTTCACGAATTTGCGTGTCAT | 107 bp |

Stem-loop RT-miR-30a-5p, Stem-loop RT-miR-30a-5p and Stem-loop RT-miR-30a-5p were used to reverse transscription of miR-30a-5p, miR-30b-5p and miR-30e-5p.

**Table S2.** Primers for the constructs.

| Name of Primers | Sequence of Primers (5′-3′) | Amplicon Size |
|---|---|---|
| MBNL1-3′ UTR-F | GCTCTAGATCAGCCACAAGACATCCACA | 448 bp |
| MBNL1-3′ UTR-R | GCTCTAGATCAGATCCCTCCCTCACCAC | |
| MBNL2-3′ UTR-F | GCTCTAGAGGGTTGTAACTGACTACAGCAT | 220 bp |
| MBNL2-3′ UTR-R | GCTCTAGAAATTGTATCGCTATTACCTTGA | |
| MBNL3-3′ UTR-F | GCTCTAGATTCAACCCGCCTAGATAGAT | 375 bp |
| MBNL3-3′ UTR-R | AAATACTGTGGAATAACCCT | |
| Pre-miR-30a-5p-F | CCCAAGCTTTTGGGAGAAGACTTAATGGTGT | 319 bp |
| Pre-miR-30a-5p-R | GGGGTACCTAATGAAAATGTAGGGATGGGT | |
| Pre-miR-30b-5p-F | CCCAAGCTTTCATGTCAATCTTTGTACCTCCTG | 291 bp |
| Pre-miR-30b-5p-R | GGGGTACCTTGCCATATCCTCTATCCGTGT | |
| Pre-miR-30e-5p-F | GGGGTACCAGGAGGAACTGAGCCGTGGACA | 241 bp |
| Pre-miR-30e-5p-R | CCCAAGCTTGACCCTGCCTGGGGACCTTTGG | |

The red font represents the sequence sites recognized by restriction enzyme.

**Table S3.** Primers for constructs of the mutant 3′ UTR.

| Name of Primers | Sequence of Primers (5′-3′) | Amplicon Size |
|---|---|---|
| Mut-MBNL1-3′ UTR-F1 | GCTCTAGATCAGCCACAAGACATCCACA | 336 bp |
| Mut-MBNL1-3′ UTR-R1 | GACCTTTGTTATTTGATTTGCTTGAAAGAAATA | |
| Mut-MBNL1-3′ UTR-F2 | TATTTCTTTCAAACAAATCAAATAACAAAGGT | 147 bp |
| Mut-MBNL1-3′ UTR-R2 | GCTCTAGATCAGATCCCTCCCTCACCAC | |
| Mut-MBNL2-3′ UTR-F | GCTCTAGAGGGTTGTAACTGACTACAGCAT | 187 bp |
| Mut-MBNL2-3′ UTR-R | GCTCTAGAAGTATTTAAAAAAGAAAACAACATT | |
| Mut-MBNL3-3′ UTR-F1 | GCTCTAGATTCAACCCGCCTAGATAGAT | 185 bp |
| Mut-MBNL3-3′ UTR-R1 | GAGCAAAAAGTTTTGTACATGTGGATTCT | |
| Mut-MBNL3-3′ UTR-F2 | AGAATCCACATGTACAAAACTTTTTGCTC | 210 bp |
| Mut-MBNL3-3′ UTR-R2 | AAATACTGTGGAATAACCCT | |

The red font represents the sequence sites recognized by restriction enzyme *Xba*I; the underline font represents the mutant sites in the target sequences recognized by miR-30-5p.

**Table S4**. Primers for real-time PCR of genes.

| Name of Primers | Sequence of Primers (5′-3′) | Amplicon Size |
|---|---|---|
| MBNL1-F | CAACAACATCTGCCACAA | 109 bp |
| MBNL1-R | TACATCTGGGTAACATACTTG | |
| MBNL2-F | CGTAACCGTTTGTATGGATTACAT | 76 bp |
| MBNL2-R | GTGTGCAGGAGGGTGAAA | |
| MBNL3-F | TGATAATACTGTGACCATCTGC | 79 bp |
| MBNL3-R | AGGAGGATGAAAATACTTGC | |
| MyoG-F | GTCCCAACCCAGGAGATCATT | 70 bp |
| MyoG-R | GACGTAAGGGAGTGCAGATTGTG | |
| MHC-F | CAATAAACTGCGGGCAAAGAC | 75 bp |
| MHC-R | CTTGCTCACTCCTCGCTTTCA | |
| MyoD-F | GGAAGGGAAGAGCAGAAG | 82 bp |
| MyoD-R | AAGGACTACAACAACAACAAC | |
| Trim55-F | AGTGAGTGGTAAGGAGTC | 97 bp |
| Trim55-R | CCAGATGTAGTAGAGAATAAGAA | |
| INSR-F | TGGAGGAGTCTTCATTCA | 100 bp |
| INSR-R | CTACTGTCCTCGGCACCAT | |
| GAPDH-F | AACTTTGGGATTGTGGAAGG | 222 bp |
| GAPDH-R | ACACATTGGGGGTAGGAACA | |

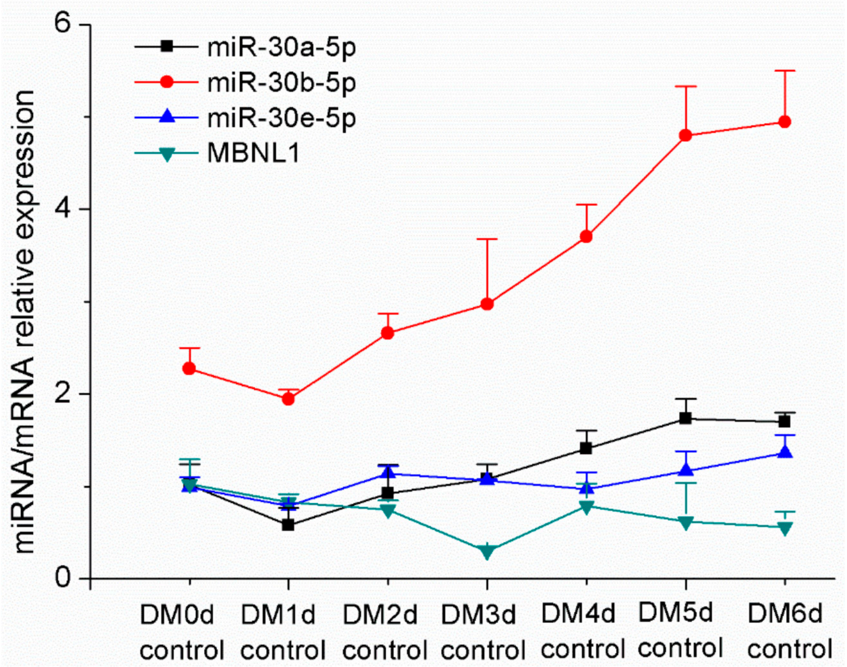


**Figure S1.** The expression of these miR-30-5p and MBNL1 in non-transfected C2C12 cells.

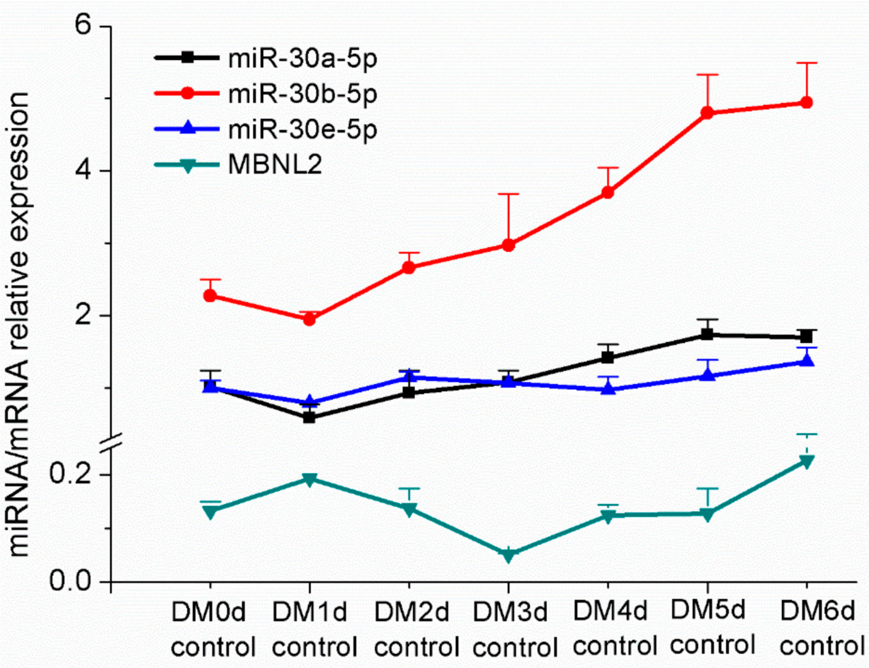


**Figure S2.** The expression of these miR-30-5p and MBNL2 in non-transfected C2C12 cells.

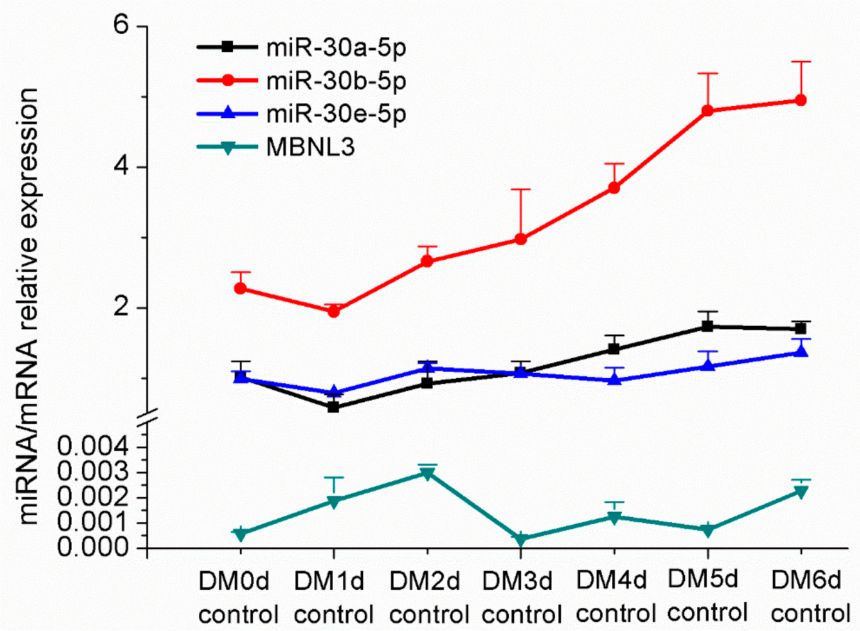


**Figure S3.** The expression of these miR-30-5p and MBNL3 in non-transfected C2C12 cells.
